# Metasurface-enhanced light detection and ranging technology

**DOI:** 10.1038/s41467-022-33450-2

**Published:** 2022-09-29

**Authors:** Renato Juliano Martins, Emil Marinov, M. Aziz Ben Youssef, Christina Kyrou, Mathilde Joubert, Constance Colmagro, Valentin Gâté, Colette Turbil, Pierre-Marie Coulon, Daniel Turover, Samira Khadir, Massimo Giudici, Charalambos Klitis, Marc Sorel, Patrice Genevet

**Affiliations:** 1 Université Cote d’Azur, CNRS, CRHEA, Rue Bernard Gregory, Sophia Antipolis, 06560 Valbonne, France; 2 NAPA-Technologies, 74160 Archamps, France; 3 Université Côte d’Azur, Centre National de La Recherche Scientifique, Institut de Physique de Nice, F-06560 Valbonne, France; 4 School of Engineering, University of Glasgow, Glasgow, G12 8LT UK; 5 Institute of Technologies for Communication, Information and Perception (TeCIP), Sant’Anna School of Advanced Studies, Via Moruzzi 1, 56127 Pisa, Italy

**Keywords:** Imaging techniques, Imaging and sensing, Metamaterials

## Abstract

Deploying advanced imaging solutions to robotic and autonomous systems by mimicking human vision requires simultaneous acquisition of multiple fields of views, named the peripheral and fovea regions. Among 3D computer vision techniques, LiDAR is currently considered at the industrial level for robotic vision. Notwithstanding the efforts on LiDAR integration and optimization, commercially available devices have slow frame rate and low resolution, notably limited by the performance of mechanical or solid-state deflection systems. Metasurfaces are versatile optical components that can distribute the optical power in desired regions of space. Here, we report on an advanced LiDAR technology that leverages from ultrafast low FoV deflectors cascaded with large area metasurfaces to achieve large FoV (150°) and high framerate (kHz) which can provide simultaneous peripheral and central imaging zones. The use of our disruptive LiDAR technology with advanced learning algorithms offers perspectives to improve perception and decision-making process of ADAS and robotic systems.

## Abstract (web summary)

Mimicking human vision with metasurfaces, the authors propose a new paradigm for high field of view and ultrafast LiDAR, achieving performances also relevant for the next generation of imaging system for ADAS and robotic systems.

## Introduction

Autonomous mobile systems such as autonomous cars and warehouse robots include multiple sensors to acquire information of their surrounding environments, defining their position, velocity, and acceleration in real time. Among them, range sensors, and in particular optical ranging sensors, provide vision to robotic systems^1–3^ and are thus at the core of the automation of industrial processes, the so-called 4.0 industrial revolution. Several optical imaging techniques are currently integrated into industrial robots for 3D image acquisition, including stereoscopic camera, RADAR, structured light illumination, and laser range finders or LiDARs. LiDAR is a technological concept introduced in the early 60 s, when Massachusetts Institute of Technology (MIT) scientists reported on the detection of echo signals upon sending optical radiation to the moon surface^4^. Since the pioneering MIT work, LiDARs have been using laser sources to illuminate targeted objects and to collect the returning echo signals offering the possibility of reconstructing highly resolved three-dimensional (3D) images. Conventional LiDARs rely on time-of-flight (ToF) measurement, which employs a pulsed laser directed toward a distant reflective object to measure the round-trip time of light pulses propagating from the laser to the scanned scene and back to a detection module. All LiDAR components must act synchronously to tag single returning pulses for ranging imaging reconstruction. The formula, $2d=c{T}_{{oF}}$, holds for the recovered distance, where $c$ is the speed of light and ${T}_{{oF}}$ is the ToF. To sense the space, the LiDAR source must be able to sweep a large Field of View (FoV). The objects in the scene are then detected, point-by-point by measuring the ToF from every single direction to build an optical echo map. The other measurement processes known as Amplitude Modulation Continuous Wave (AMCW)^5,6^, Frequency Modulation Continuous Wave (FMCW)^7,8^ or Stepped Frequency Continuous Wave (SFCW)^9^ employ continuous waves with constant or time-modulated frequency to measure the round-trip time of the modulated light information. LiDAR systems enable the real-time 3D mapping of objects located at long, medium or short-range distances from the source, finding a vast variety of applications beyond robotic vision, spanning from landscape mapping Chase^10–12^, atmospheric particle detection^13–16^, wind speed measurements^17,18^, static and/or moving object tracking^19–22^, AR/VR^23^, among others. Generally, LiDARs are classified into scanning or non-scanning (Flash LiDAR) systems depending on whether the laser sources simply illuminate^24^ or scan the targeted scene. A scanning LiDAR system can be essentially described in terms of three key components, (i) the light source for illumination, (ii) the scanning module for fast beam direction at different points in the scene, and (iii) the detection system for high-speed recovering of the optical information received from the scene. Over the past decades, nanophotonics-based LiDAR systems have blossomed, and more advanced scanning and detection techniques have been proposed^25,26^. The expected massive use of LiDARs in the automotive industry for advanced driver-assistance systems (ADAS) or even full-autonomous driving brought out new challenges for the scanning systems, including low fabrication complexity, potential for scalable manufacturing, cost, lightweight, tolerance to vibrations and so on. Today, industrially relevant LiDARs mainly use macro-mechanical systems to scan the entire 360° FoV. Besides their large FoV, these bulk systems present limited imaging rates of the order of few tens of Hz. A promising evolution in mechanical scanners are the micro-electromechanical systems^27^ (MEMS) which shift the scanning frequency to the kHz range. However, a major drawback of MEMS is the low FoV, typically not exceeding 25° for horizontal and 15° for vertical scanning. At the research level, beam steering with optical phased arrays (OPA)^28,29^ provides remarkable speeds while reaching FoV around 60°. However, OPA technology is less likely to be massively deployed in industrial systems due to its manufacturing challenges. The industrially mature liquid crystal modulators are also not adequate as LiDAR scanners due to their poor FoVs usually remaining below 20° depending on the wavelength, as well as their kHz modulation frequency^30,31^. Moreover, acousto-optic deflectors (AODs) enabling ultrafast MHz scanning^32,33^, have never been considered in LiDARs because of their narrow FoV reaching at maximum 2°, imposing a compromise between high-speed imaging and large FoV.

During the last decade, metasurfaces (MS)^34^ have spurred the interest of the entire international photonic community by unveiling the possibility of engineering the properties (i.e., the amplitude, the phase, the frequency and/or the polarization) of light at will^35^. They are flat optical components made of arrangements of scattering objects (meta-atoms) of subwavelength size and periodicity. Currently, four light modulation mechanisms are used to create metasurfaces: light scattering from resonant nanoparticles^36,37^,geometric phase occurring during polarization conversion (Pancharatnam–Berry phase)^38^, accumulated propagation phase in pillars with controllable effective Refractive Index (ERI)^39^ and the topological phase in vicinity of singularities^40^. Usually, MSs comprise inherently passive components, designed to perform a fixed optical functionality after fabrication. For instance, by properly selecting the size and the spacing of the meta-atoms, MSs allow to redirect a laser beam at any arbitrary but fixed angle dictated by the generalized Snell’s law. Clearly, passive MS alone cannot be used in LiDARs requiring real-time beam scanning. On the contrary, dynamic MSs designed by—or combined with—materials possessing tunable optical properties caused by external stimuli^41–45^ stand as promising alternatives for real-time deflection. Recently, the US startup company LUMOTIVE introduced electrically addressable reflective resonant MSs infiltrated with liquid crystals and demonstrated scanning frequency that exceeds the switching speed of common liquid crystal displays, as well as a FoV of around 120°^46^. The latter approach has been proven auspicious for miniaturized, scalable LiDARs but it involves complex electronic architectures, and likely significant optical losses in case of metallic MS building blocks.

Here, we propose an alternative high-frequency beam scanning approach that exploits the light deflecting capabilities of passive MSs to expand the LiDAR FoV to 150 × 150°, and to achieve simultaneous low- and high-resolution multizone imaging. We make use of an ERI multibeam deflecting MS cascaded with a commercial AOD. The system offers large flexibilities in terms of beam scanning performance, operation wavelength and materials. The angular resolution, referring to the ability of the system to distinguish adjacent targets and retrieve shapes, becomes very important in applications requiring simultaneous long and short-range detections. Our multizone LiDAR imaging demonstration can mimic human vision by achieving simultaneous high frame rate acquisition of high- and low-field zones with different spatial resolution. The large design flexibility of MSs provides imaging capabilities of interest to LiDAR systems, meanwhile offering new industrial applications.

### Ultrafast and high-FoV metasurface scanning module

**Fig. 1:**
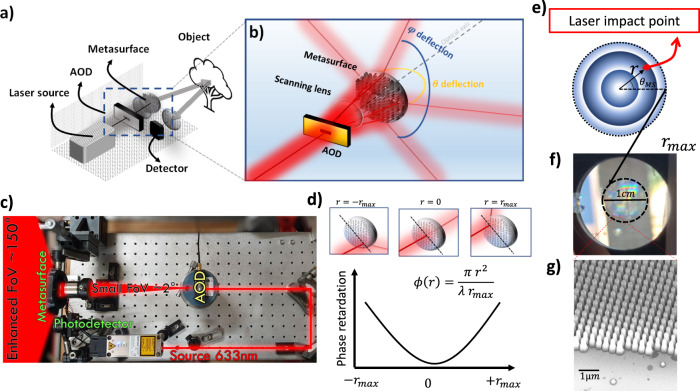
Concept of a metasurface-augmented FoV LidAR. **a** Schematic representation of the LIDAR system. A triggered laser source, emitting single pulses for ToF detection, is directed to a synchronized acousto-optic deflector (AOD) offering ultrafast light scanning with low FoV (~2°). The deflected beam is directed to a scanning lens to scan the laser spot on the metasurface at different radial and azimuthal positions. The transmitted light across the metasurface is deviated according to the position of the impinging beam on the component to cover a scanning range between $-75^\circ$ and $75^\circ$. The scattered light from the scene is collected using a fast detector. Data are processed to extract the single echo ToF for 2D and 3D imaging of the scene. **b** Detail of the cascaded AOD-metasurface assembled deflection system. **c** Top view photography of the optical setup. **d** Bottom: Graphical representation of the metasurface phase distribution along the radial axis. Top: Representations of beam deflection according to the incident beam positioning on the metasurface. Inset equation represents the phase function designed. **e** Illustration of axial symmetry for the laser impact point. **f** Photography of the 1 cm MS fabricated using nanoimprinting lithography. **g** SEM image of the sample showing the nanopillar building blocks of varying sizes employed to achieve beam deflection by considering lateral effective refractive index variations.

MHz beam scanning can be achieved over a large FoV, by coupling AODs with ERI MSs exhibiting spatially varying deflection angles. Figure 1a illustrates the experimental concept where a modulated laser source at $\lambda=633\,{{{{{\mathrm{{nm}}}}}}}$ (TOPTICA i-beam smart) generates single pulses at any arbitrary rate up to $250\,{{{{{\mathrm{{MHz}}}}}}}$. For single-pulse LIDAR, the repetition rate ${f}_{{rep}}$ is related to the maximum ranging distance ${d}_{{\max }}$ by the expression:

1 $${d}_{{\max }}=\frac{1}{2}\frac{c}{{f}_{{rep}}}.$$

The focused beam with a small deflection is angularly increased to scan in both azimuthal $\theta$ and elevation $\varphi$ angles. A detailed scheme of the FoV amplifying system is shown in Fig. 1b. A photograph of the built proof-of-concept system is shown in Fig. 1c where we highlighted (shaded red region) the expansion of the small two degrees (2°) AOD FoV into an enhanced 150° FoV. The deflected angle by the MS is controlled by the impact position of the impinging focused beam on the MS plane, associated with the radial and angular coordinates $r$ and ${\theta }_{{MS}}$, respectively (see Fig. 1e). By applying voltage into the AOD, one can actively re-point the beam at any arbitrary angle within the 2° × 2° FoV, thus sweeping the focused beam across the metasurface to vary ${\theta }_{{MS}}$ and $r$, in the range of [$0-2\pi$] and [$0-{r}_{{\max }}$], respectively, where ${r}_{{\max }}$ is the radius of the metasurface. Note that ${\theta }_{{MS}}$ and $r$ denote, in polar coordinate, the position of the impact beam on the metasurface according to Fig. 1e. For simplicity in connecting incident and deflected angles, we designed a circular metasurface with radially symmetric phase-delaying response, but given the versatility in controlling the optical wavefront, various MS with any other beam defecting properties can be adjusted according to specific application. We must also highlight that, in principle, there is no limitation on the observed FoV as it is fully dependent on the metasurface phase function, within the limit $[0,\;\pi ]$ for transmission scheme. In this initial demonstration, we implemented (Fig. 1f, g) the simple concept of ERI MS designed to spatially impart linearly increasing momentum with respect to the radial dimension $r$ given by the expression:

2 $$\frac{\partial \Phi }{\partial r}=-{k}_{0}\frac{r}{{r}_{{\max }}}$$

where, ${k}_{0}$ is the free space momentum, and $\Phi$ the local-phase retardation. Such design results in parabolic-phase retardation as represented in Fig. 1d. In this design, the deflected beam will be delayed by a maximum phase retardation of $\Phi=\mp \frac{\pi {r}_{{\max }}}{\lambda }$ and $\Phi=0$ for the peripherical points $\pm {r}_{{\max }}$ and central points, respectively. Moreover, Eq. (2) transformed in Cartesian coordinates determines the value of the deflected angles in both axes, denoted as $(\theta,\;\varphi )$, according to the generalized Snell laws^41^:

3 $$\left\{\begin{array}{c}{k}_{x,t}={k}_{x,i}+\frac{\partial \Phi }{\partial x}=\,{k}_{0}{{\sin }}{\theta }_{i}{{\sin }}{\varphi }_{i}+\,\frac{\partial \Phi }{\partial r}\frac{\partial r}{\partial x}\\ {k}_{y,t}={k}_{y,i}+\frac{\partial \Phi }{\partial y}={k}_{0}{{\sin }}{\theta }_{i}{{\cos }}{\varphi }_{i}+\,\frac{\partial \Phi }{\partial r}\frac{\partial r}{\partial y}\end{array},\right.$$

where the phase gradient is defined at the metasurface plane at $z=0$. Considering small incident angles originating from the AOD, the expressions simplify as:

4 $$\left\{\begin{array}{c}{k}_{0}{{\sin }}{\theta }_{t}{{\sin }}{\varphi }_{t}=\,-{k}_{0}\frac{r}{{r}_{{\max }}}{{\cos }}{\theta }_{{MS}}\\ {k}_{0}{{\sin }}{\theta }_{t}{{\cos }}{\varphi }_{t}=-{k}_{0}\frac{r}{{r}_{{\max }}}{{\sin }}{\theta }_{{MS}}\end{array}\right.\,,$$

Such expression validates the linearity observed for small angles [−40°, 40°] according to the experimental measurements of the voltage dependence of the deflection angles (Supplemental Fig. S2c).

## Results

### 2D and 3D LiDAR image acquisition

**Fig. 2:**
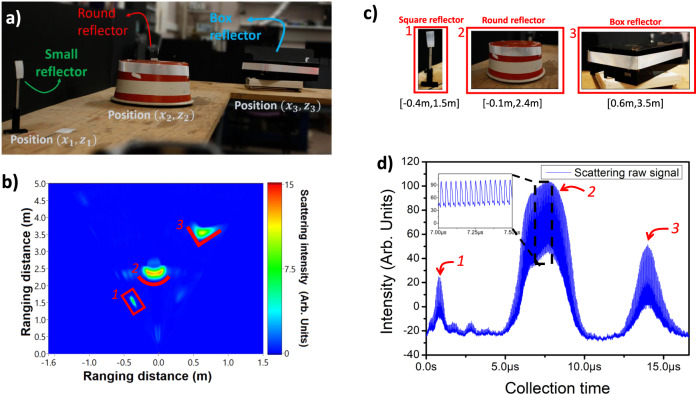
1D time-of-flight imaging. **a** Photography of the scene. **b** Ranging image of three objects displaced on a table using high reflective tapes to improve the intensity of the returned signal. In (1) a post with a small reflector was used in (2) a round object with a reflector and in (3) there is a box reflector with a tape around it. The graph shows the image in the correct ranging distance *X* (scanning dimension) and *Z* (ranging dimension) showing the capabilities to sense all of the three objects. **c** Position of single objects according to ranging image in (**b**). **d** Raw signal collected for the respective image, showing that objects oriented in the normal direction have bigger scattering intensity, the inset display single pulses used to determine the ToF ranging distance.

To show the angular and depth 2D imaging capabilities of our LIDAR system, we start performing 1D scanning of three distinct objects placed on a table, (1) a square reflector mounted on a post, (2) a round deflector and (3) a box reflector, angularly distributed at different locations as shown in Fig. 2a. The associated 2D LIDAR ranging image is displayed in Fig. 2b, indicating that high reflectivity objects are observed at LIDAR positions matching to those observed with a conventional camera (Fig. 2a). Particularly, we found that the three objects shown in Fig. 2c were located at the following width [*x*], and depth [*z*] positions: [−0.4 m, 1.5 m], [−0.1 m, 2.4 m] and [0.6 m, 3.5 m] for the square, the round, and the box reflector, respectively. In the graph, we also observe the difference in reflectivity of the three objects at various distances leading to distinct intensities: the objects on the left and right (square and box deflectors) correspond to lower signals due to their angular locations, size and distance, while the round deflector in the middle has higher reflectivity and appears with higher reflectance. This first example validates the short-range (~5 m) imaging capabilities of our LiDAR system.

**Fig. 3:**
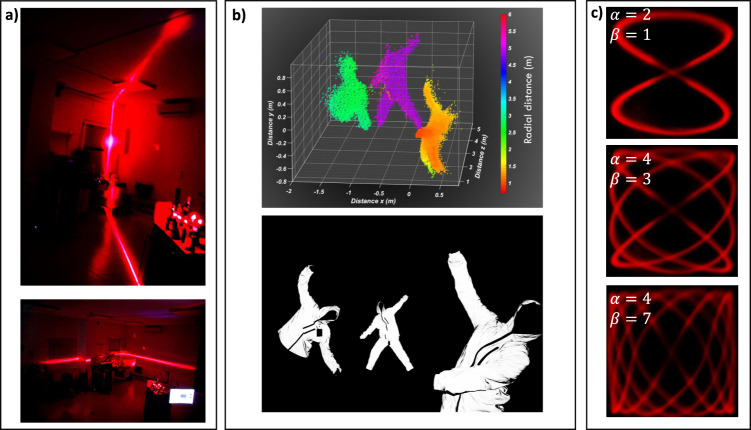
3D imaging and wide-angle scanning capabilities. **a** LIDAR line scanning of our laboratory room that show the large FoV on both Elevation (top) and Azimuth (bottom) angles. Note the top picture showing a scanning line profile covering the whole range from the ground to the ceiling of the testing room over 150°. **b** 3D ranging demonstration (top): the scene (bottom) was set up with actors wearing reflective suits positioned in the scene at distance *Z* varying from 1.2 to 4.9 m. Colors encodes distance. **c** Lissajous scanning using deflecting functions as $\theta=A{\sin }\left(\alpha t+\varPsi \right)$ and $=B{\sin }\left(\beta t\right)$ for different parameters $\alpha$ and $\beta$ to illustrate the laser projection capabilities on a fast beam scanning, in a large FoV configuration. Ψ was set to be 0° and *A* = *B* = 30, although any configuration can be actively changed.

To further investigate the capabilities of the system, we extended the performance to achieve 3D imaging. To this end, an additional FoV dimension is added by cascading a second AOD, orthogonally oriented, in the elevation axis. The extended FoV is now improved over both dimensions considering a MS with radial symmetry, as schematized in Fig. 1b. To demonstrate the two-axis scanning capability, we present in Fig. 3a the elevation (top) and the azimuthal (bottom) line scanning, respectively, to highlight that 150° FoV (Supplementary Materials S1) is accessible for both scanning axis (see video V1 in supplement materials). These examples of line scanning are realized by fixing the voltage value on the one deflector and scanning the voltage of the second deflector over the entire range at a scanning rate that exceeds the acquisition speed of either our eye or the CCD refreshing frame rate, resulting in an apparent continuous line scan. We prepared a scene (Fig. 3b)—bottom) with three different actors located at different angular and depth positions of 1.2, 2.7, and 4.9 m to demonstrate 3D imaging. Due to low laser pulse peak power (about 10 mW), we performed our demonstrations in an indoor environment using high reflective suits, considerations of power and losses are addressed in Section S2 of Supplemental Materials. For the demonstration, we choose a visible laser operating at $\lambda=633\,{{{{{\mathrm{{nm}}}}}}}$, which is very convenient to observe and monitor the deflected beam. After calibrating the system (see Supplementary Material), arbitrary—or random access—beam scanning along high FoV can be realized and arbitrary intensity patterns can be projected by rapidly steering the beam at different locations at very short time intervals (see Supplementary Video V2). Figure 3c shows examples of several scanning profiles implemented to the metasurface beam scanner to project Lissajous curves demonstrating random-point access mode.

### Mimicking human peripheral and fovea vision with multizone LiDAR imaging

**Fig. 4:**
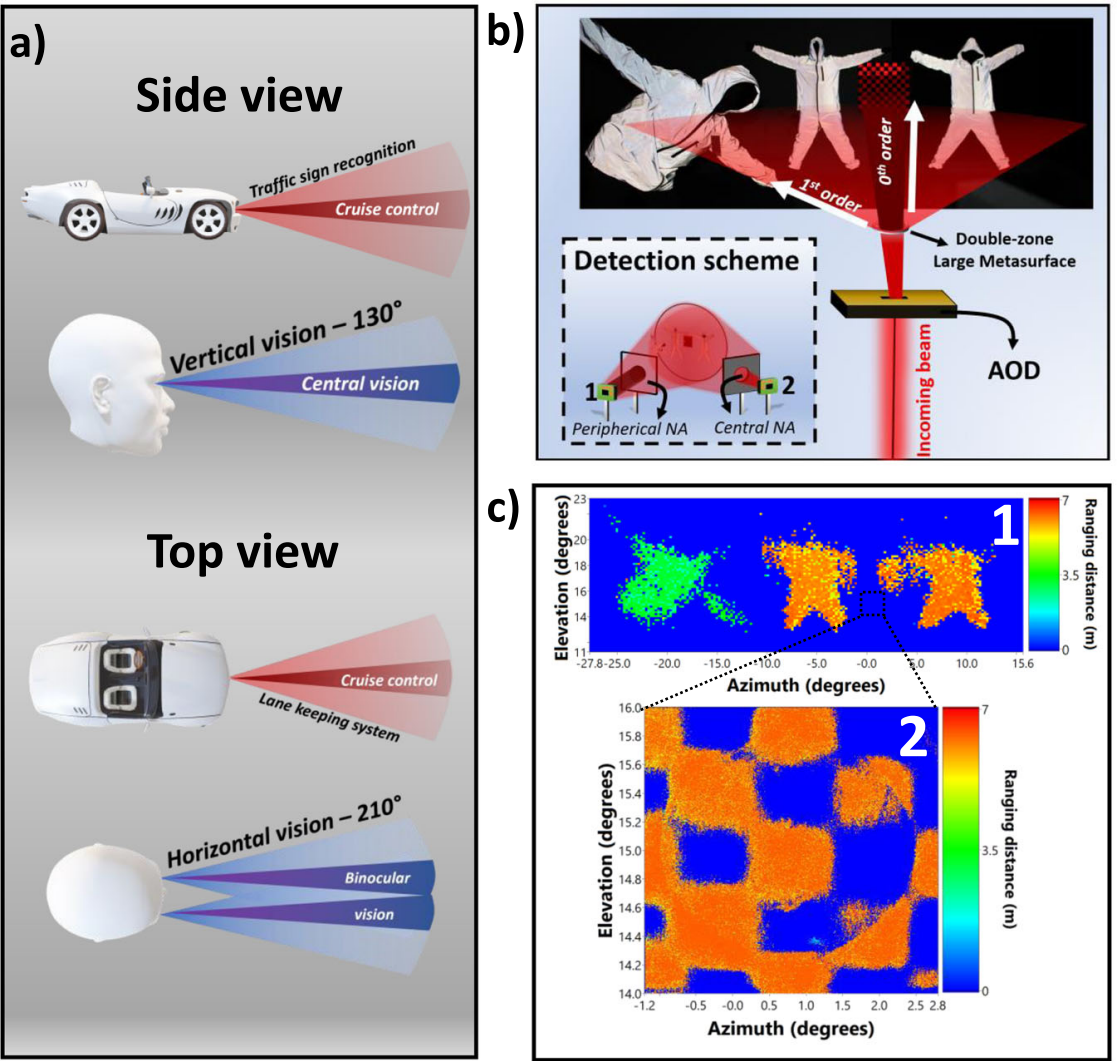
Multizone imaging. **a** Schematic representation of a human multizone viewing with the concept to be adapted in ADAS systems. Such mimicking characteristics enables double vision for dual-purpose imaging features for high-resolution, long range, in the center and lower resolution, bigger FoV, for the peripherical view. **b** Experimental realization to test the dual-zone imaging functionality of the LIDAR system, including dual detection scheme (inset) for simultaneous image multiplexed collection. The central 0th diffraction order beam scans a small area with high resolution directed at the center of the image while the 1st diffracted order scans the whole field. **c** Top: We show the result of the scanned scenes described in (**b**). Top represents the LIDAR large FoV ranging image. The image is obtained by blocking the central part of the numerical aperture using an obstacle as sketched in (**b**). The bottom LIDAR ranging high-resolution image presents the central part scene captured using the 0th diffraction beam, covering a FoV of about 2°.

Previous experiments were performed by focusing the light deflected by the AOD on relatively small metasurfaces (1, 2, and 3 mm diameters) using a scanning lens. This configuration favors a small spot (of the order of 50 μm) to contain the MS angular divergence to a small parametric region, i.e., scanning the MS with small spot prevents large overlap with the spatially varying deflecting area. The beam divergence as a function of the metasurface size is provided in Supplementary Material S5, indicating that a 3 mm device results in a divergence lower than 1.5°. Robotic systems interested in reproducing human vision requires peripheral and central vision as illustrated in Fig. 4a, where several zones featuring different spatial resolutions are acquired simultaneously. A low-resolution peripheral field provides coarse scene exploration, usually needed for human to direct the eye to focus to a highly resolved fovea region for sharp imaging. The scene thus needs to be scanned differently according to the zones of interest. To reduce further beam divergence and improve as needed the resolution, it is necessary to increase the diameter and complexity of the metasurface and work with fully collimated beams. For this purpose, we realized a cm-size metasurface deflector using nanoimprint lithography (NIL), as shown in Fig. 1f, g (further details on the fabrication are provided in S9). In the latter configuration, the deflector is directly placed after the AOD without utilizing a scanning lens. We specifically designed a large area deflector that achieve moderate 1st order deflection efficiency of ~40% and took advantage of the non-deflected zero-order narrow scanning FoV to simultaneously scan two zones with different FoVs and resolutions. This demonstration specifically exploits the multibeam addressing capability of metasurfaces, resulting in a dual mode imaging: (i) a high-resolution scanning provided by the near collimated zero-order beam deflected by the AOD only, and (ii) a large FoV, lower resolution image provided by the 1st order beam deflected by the metasurface. As illustrated in Fig. 4b, inset, we spatially selected the returned/scattered signal from the different parts of the scene. For this purpose, we used a double-detector monitoring scheme. The first detector collects light from the full numerical aperture (~2$\pi$ solid angle) but it blocks the central small numerical aperture (a beam blocker is placed in front of the detector). The second detector covers only a small NA for the narrow FoV resulting from zero-order light scanning (a spatial filter is used to select the observation area). A dual-beam metasurface scanning scheme is used to image a scene (Fig. 4b, top) with two fields of interest: (i) three actors placed at different regions of the space periphery, as measured in Fig. 4c (top) and a highly resolved chessboard-like object placed in the forward direction at a small FoV, measured in Fig. 4c (bottom). The images presented in Fig. 4c correspond to low- and highly resolved imaging, acquired by both detectors simultaneously. Multizones scanning with a high resolution forward, and low lateral resolution over a high-FoV peripherical vision could be a disruptive solution for addressing the needs of advanced driver-assistance systems (ADAS).

### High-speed velocimetry and time-series imaging

To characterize the MHz deflection speed and the possibility of achieving real-time frame rate imaging, we measured the beam deflection speed, i.e., the minimum frequency at which the beam can be re-pointed to a new direction. To do so, we placed highly reflecting tapes on the wall, and measured the amplitude of the backscattered signal for distinct scanning frequencies. We define as “system cutoff frequency” the condition when the amplitude of the reflected signal decays to −3dB point (see Supplementary Information S4). The measurements were made by considering: (i) a single scanner in the azimuth angle (see Supplementary Fig. S5b red curve) and (ii) a cascaded system comprised by two orthogonally oriented deflectors, for scanning at both azimuthal and elevation angles, (see Supplementary Fig. S5b) blue curve). The results indicate less than −3 dB loss up to around 6 MHz and 10 MHz for single and double-axis scanning, respectively. We also demonstrate the modulation of a laser beam over an large FoV (>140°) at MHz speed and correct imaging with scanning frequency up to 6.25 MHz (see Supplemental Fig. S5c)). This corresponds to about two orders of magnitude faster than any other beam-pointing technology reported so far. Operating beyond the −3 dB loss at higher frequency was also realized, leading to reduced resolution but increased imaging frame rate, up to 1 MHz for 1D scanning at 40 MHz (see discussion in Supplementary Materials in Section S7).

**Fig. 5:**
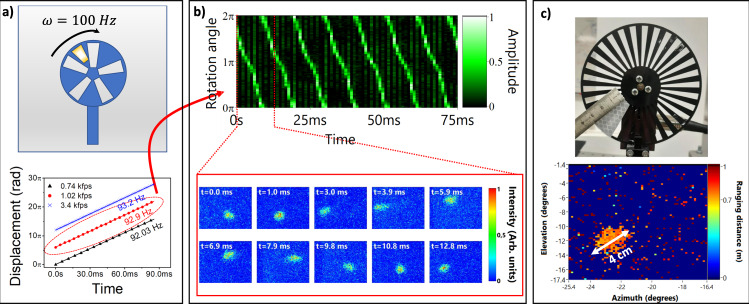
Measurement of fast in real-time-series events. **a** Top: Illustration of the scene: a mechanical chopper of was set up with a nominal speed of 100 Hz and some slabs were covered using a reflective tape. Bottom: Measurement of the rotation speed for three different frame rates. **b** Top: Normalized intensity map for the radial axis, illustrating the dynamics of the wheel. Note the different slope for the rotation angles around $3{{\pi }}/2$ representing a lessening of the speed. Bottom: Single-frame intensity data illustrating various angular positions. **c** Top: photography of the chopper and the size of the reflective tape. Bottom: Ranging image for *t* = 1.0 ms and the measurement of the tape from the recovered data.

Measurements of time events were performed to investigate dynamic imaging. The most convenient dynamic system observable in our laboratory was a spinning chopper composed of a rotating wheel at nominally 100 Hz rotation speed. We prepared the scene composed of a chopper, located at 70 cm away from the source, decorated with a high reflective tape in one of the mechanical shutters, as illustrated in Fig. 5a (top). As described in Supplemental Table 1, we performed three time-series experiments using acquisition frame rates of 741, 1020, and 3401 fps (see Supplementary Information S8 and Supplementary Videos SGIF 1–3). We tracked the center position of the reflective tape in both the space and time domains by integrating the radial axis of the ranging image from the center of the chopper and fitting a Gaussian curve plotted over the entire $[{{{{\mathrm{0,2}}}}}\pi ]$ angular axis (see Fig. 5a, bottom). The curves are manually offset by 6$\pi$ to differentiate the experiments. All experiments revealed an averaged rotation speed value of 92.71 Hz. We attribute the ~7.3 Hz difference between the measured and nominal speed of 100 Hz to the phase-jitter control mechanism on the chopper. In principle, rotating mechanical shutters are designed with a closed loop circuitry providing an electronic signal that maintains linear rotation speed. Interestingly, displaying time events on the angular dimension reveals small wobbling wheel imperfection caused by the presence of the reflective tape, resulting in a slowdown at the angles around $3\pi /2$ as evidenced in Fig. 5b (Experiment 2—1020 fps). One can indeed observe a rotation slope-change during periodic times corresponding to the position of the reflective tape at the bottom (for instance at *t* = 1.0 ms/10.8 ms in Fig. 5b) (bottom panel)). Using the recovered ranging information, we estimate the size of the tape to 4 cm, as illustrated in Fig. 5c (bottom). The 1 cm difference to the real object (Fig. 5c, top) is due to the high reflectivity of the screws located close to the center and causing additional scattering at the same ranging distance.

## Discussion

We realize an ultrafast beam scanning system composed of a fast deflector and a passive metasurface to achieve beam steering at MHz speed over 150 × 150° FoV, improving the wide-angle scanning rate of mechanical devices by five orders of magnitude. We performed fast steering in one and two angular dimensions and retrieved the associated time of flight for ranging measurements leading to high-speed LiDAR imaging of very fast-moving objects on a large FoV. Employing parameters described on the second row of Supplementary Table 1, we achieved a time step of 980 µs, see Fig. 5b. An object traveling at the speed of the sound (1234 Km/h) at 15 m away from the source will take ~74 ms to cover a 120° FoV. Such supersonic object can be detected within 76 time-series events. Considering the Nyquist limit i.e., four time series to recover the speed, the maximum event detection can increase up to a speed of 47 mega-meter/h.

High-speed scanning modules for LiDAR applications have to trade-off between the maximum distance and spatial resolution (see Supplementary Information S5, S10). The frame rate of a single ToF system can be expressed as:

5 $${f}_{{Rate}}=\frac{c}{2\,n\,{d}_{{\max }}}$$

where $c$ is the speed of the light. Equation (5) thus indicates that both the number of pixels in the image ($n$) and the maximum ambiguity distance, $({d}_{{\max }})$, defines the imaging frame rate. Such echoing time can be reduced by encoding the signal sent in each scanning direction with a specific identification code namely Code-division multiple access (CDMA)^47^. Multiplexed observation is realized by decorrelating the ToF signal using matched filter technique. LiDAR companies often multiplex the source with an array of diode lasers to increase frame rate, increasing the lidar complexity, and multiplying the system cost by the number of sources. Such CDMA technique realistically could be exploited in combination with our fast beam deflection system to reach imaging frame rate of 125 frames/s with high spatial resolution of 200 × 200 pixels. Beyond application for ADAS industry, beam steering systems with similar performances have potential in real-time imaging for applications requiring short ambiguity distance, for example in microscopy and wide-angle optical coherence tomography^48^. Our main limitation to achieve high frame-rate is related to the extremely large volume of real-time data treatment to be realized synchronously during the acquisition. Here we only performed calculation using conventional CPU—LabView based—as such, we cannot output and save data as the same speed as their acquisition. The Supplementary Video V3 showing a moving person in 3D space is taken by achieving the best compromise, that is by acquiring single frames raw data (with 200 × 200 pixels for instance) and outputting data directly to SSD driver frame-by-frame. Our data treatment process creates latencies related to asynchronous data storage, which result in stuttered or choppy movements with occasional video speeding-up movements. This problem is generally mitigated in LiDAR by implementing FPGA/ASICS processing.

Our approach also offers random-access beam steering capabilities. Multizone ranging images mimicking human vision at high frame rate have been realized. The versatility of MS for wavefront engineering could improve the capabilities of simultaneous localization and mapping algorithms. Furthermore, incorporating this system in ADAS could provide a disruptive solution for medium/long-range perception, in which the central view scans the front scene, while the peripheral view provides additional sensing for pedestrian safety for example. We finally demonstrated time-event series for imaging at a real-time regime (>1k fps and up to MHz frame rate for 1D scanning). Outperforming existing LiDAR technologies, our tool offers a perspective for future applications, in particular by participating to reducing the low decision-making latency of robotic and advanced driver-assistance systems.

## Methods

### Experimental methodology

A collimated beam is sent to an AOD device (AA Opto-electronic DTSXY-400-633) to deflect light at small arbitrary angles, within 49 *mrad*. The AOD is driven by a voltage-controlled RF generator (AA Opto-electronics DRFA10Y2X-D-34-90.210). The deflected signal is directed to a scanning lens (THORLABS LSM03-VIS) that focuses the light at different transverse positions on the MS. The MS acts as a designer-defined passive device to convert the small 2° × 2° FoV into an enhanced 150° × 150° FoV. ToF is obtained by monitoring the scattered light at each scanned angle using a detector (Hamamatsu C14193-1325SA); and the reconstructed ranging image is built by associating each period $(\frac{1}{{f}_{{rep}}})$ to individual pixels and extracting the ToF. In our detection scheme, the detection path is separated to the excitation path, which may result in not overlapped illumination/observation regions. We believe that a mono-static approach could as well be implemented in our configuration by utilizing a beam splitter before sending the laser beam into the acousto-optics deflector. A PXI (National Instruments) system is used for data generation, recovery, and treatment (more details can be found in Section S6 of Supplemental Materials). The angular scanning of the whole 1D was performed in a single shot, during which we orchestrated pulse repetition, scanning position angles and collection for precise measurement of ToF in the system. With an acquisition scope card of 3Gsamples/s sample rate and considering a rise time on the detector smaller than ~330 ps, the maximum z (depth) resolution of single echo per laser shot measurement is about $\Delta z=5{cm}$. In Fig. 2d, we show the collected raw signal corresponding to the three objects. For ToF recovery, we used the derivative of the signal and collected the peak of the differentiated signal. Single pulses were collected (inset Fig. 2d) and separated to evaluate the ToF for each scanned direction and then folded at the scanning frequency to form an image. The fabrication of the different MS has been realized using GaN on sapphire nanofabrication processes. Details are available in the supplementary materials.

## Supplementary information

Supplementary Information

Peer review file

Description to Additional Supplementary Information

Supplementary GIF 1

Supplementary GIF 2

Supplementary GIF 3

## Data Availability

The Source data are available from the corresponding author upon request. All data needed to evaluate the conclusion are present in the manuscript and/or the Supplementary Information. Videos are available as Supplementary Materials, and the associated raw data would be available upon request.
