# Supplementary material for: Metasurface-enhanced light detection and ranging technology
Source: Nat Commun. 2022 Sep 29;13:5724. doi: 10.1038/s41467-022-33450-2 (PMC9523074; doi:10.1038/s41467-022-33450-2)
Supplement: Supplementary file 1 — Supplementary Information [file 41467_2022_33450_MOESM1_ESM.pdf]

Supplementary Information

Authors

Renato Juliano Martins[1], Emil Marinov[1], M. Aziz Ben Youssef[1], Christina Kyrou[1], Mathilde Joubert[1], Constance Colmagro[1,2], Valentin Gâté[2], Colette Turbil[2], Pierre-Marie Coulon[1], Daniel Turover[2], Samira Khadir[1], Massimo Giudici[3], Charalambos Klitis[4], Marc Sorel[4,5] and Patrice Genevet[1†]

Affiliations

[1] Université Cote d'Azur, CNRS, CRHEA, Rue Bernard Gregory, Sophia Antipolis 06560 Valbonne, France

[2] NAPA-Technologies, 74160 Archamps, France

[3] Université Côte d'Azur, Centre National de La Recherche Scientifique, Institut de Physique de Nice, F-06560 Valbonne, France

[4]School of Engineering, University of Glasgow, Glasgow, G12 8LT, UK

[5]Institute of Technologies for Communication, Information and Perception (TeCIP), Sant'Anna School of Advanced Studies, Via Moruzzi 1, 56127, Pisa, Italy

† Corresponding Author: Patrice.Genevet@crhea.cnrs.fr

LiDAR and beam steering videos are available on the Youtube Channel here:

https://www.youtube.com/channel/UCmezaBH-xOxMjqk3bvqnlAg

S1. Beam deflection characterization

A solid calibration procedure which correlates the deflection angle with the voltage applied into the AOD RF driver enables reliable beam scanning with low image distortion. The results shown in figure S1a) demonstrate deflection angles varying from -60 to 60 degrees for AOD voltage actuation ranging from 0 to 10V on the 2D deflection system (AA Opto-electronic DTSXY-400-633). The calibration curve in figure S1c) indicates a total FoV of 150 degrees and the green lines denote the absolute maximum deflection angle before the beam shape starts being deformed, accompanied with significant efficiency drop. The displacement of the impact spot on the MS is a function of both the AOD deflection angle and the distance between the AOD and the MS. For calibration purposes and to account for the radial symmetry of the MS, we experimentally constrained the system such that deflections in the range -90 and +90 degrees correspond to +5 and –5 AOD voltage respectively. This 180-degree interval corresponds to the maximum FoV predicted by the sample design. However extreme FoV deflection are not observed in the experiments due to low MS efficiency at extreme angles. If this issue can be addressed by employing a MS designed for high deflection angles. The experimental points were fitted by a third order polynomial function, which provides a continuous and analytical expression allowing global calibration for any arbitrary scanning. Results are shown in Figure 3c) and analytically described hereafter in section S3.

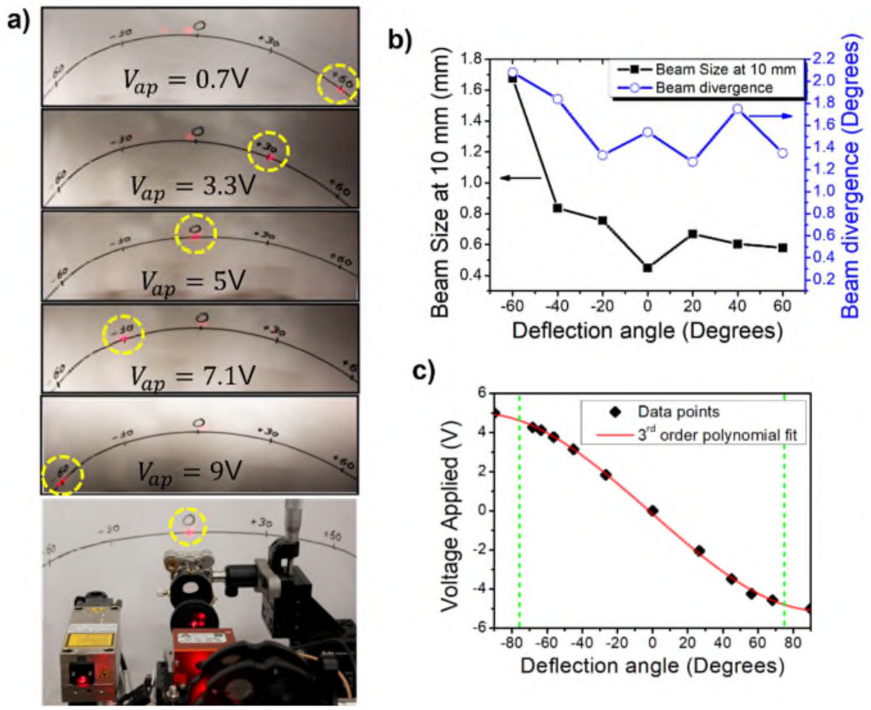

Figure S1 - Deflection capabilities – In **a)** we illustrate the beam deflection as function of the applied voltage ranging from 0.7V to 9V, by employing the AOD driver. One can observe some faint small orders of diffraction leaking from the metasurface due to fabrication imperfections, as well as to the overlay of high orders of deflected beam from the metasurface. In the bottom we illustrate the reference of the LIDAR point-of-view. In **b)** we present the beam size measurement as a function of the deflection angle to characterize the beam divergence. In **c)** we show the calibration curve as function of the AOD applied voltages. We aligned the system such that the angle of 0 degrees correspond to 0V. This approach allows us to analytically fit the calibration axis of symmetry, R, represented by red curve. Green dashed traces represent the limits in which we were able to measure the deflection; below and above those points important beam distortion occurred and no angle could be accurately measured.

Moreover, as plotted in fig. S1b), we investigate the beam shape (divergence and size) for several deflection directions, in the range of [-60°, +60°]. These measurements determine the maximum achievable spatial resolution. They also quantitatively show the evolution of the imaging quality for increasing deflection angles. Low divergence is observed when the beam is directed towards the MS center. Working with a scanning lens, i.e. focused beam instead of collimated wavefront, imposes additional distortions. We found a variation in the beam divergence between 1.4 and 2.0 degrees for the beam passing at the center and at -60°, respectively (blue curve, right axis on fig. S1b). The beam waist size increases by around 4 times at -60° due to previously mentioned effects. Note that due to MS design and fabrication imperfections, we observe the presence of a small residual zeroth diffraction order located around 0 degree.

S2. Losses and power distribution

Efficient and low-noise LiDAR sensing requires high power sources to illuminate the scene at a wavelength for which the environment does not add significant absorption/scattering losses. LiDARs utilized for ADAS and/or robotic systems usually operate at IR wavelengths of 905nm/940nm and 1550nm. Prototype system must in principle rely on these wavelengths due to the atmospheric absorption and the lower solar background. Here we are proposing a system operating at conventional visible wavelength for proof of concept demonstration. Visible wavelengths are not optimal for LiDAR but allow an easy visualization of

the scanning path. Due to the easy scaling properties of MS, this concept can similarly be exploited at IR frequencies. To characterize the typical optical losses on the modules constituting the system described in Figure 1, we considered all the loss channels, including deflection efficiency of both AOD and MS.

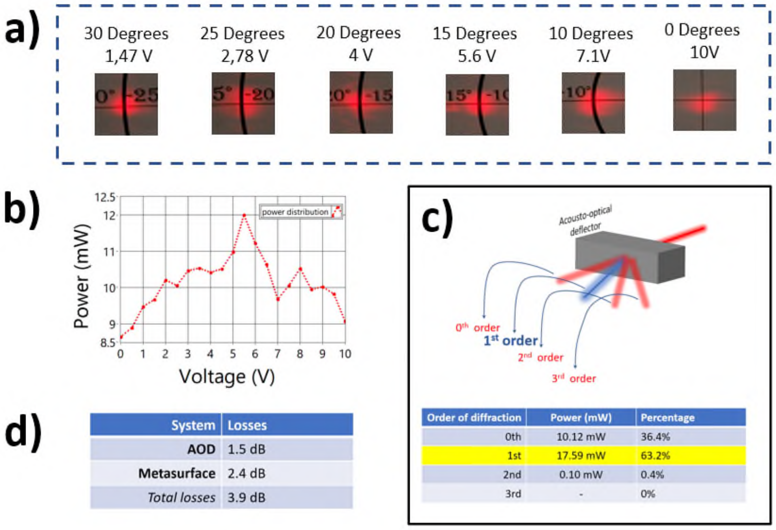

Figure S2 – Losses measurements – **a)** Beam profile in function of the deflection angle and the correspondent voltage. When the beam moves towards higher angles, we observe a distortion in the shape evidenced by an elongated shape, at 10V. **b)** Distribution of laser power according to the deflection angle. **c)** Power distribution of the diffracted orders on the AOD when optimizing for the 1st order. **d)** Overall losses measured on the final LiDAR system accounting both lossy AOD and MS.

In figure S2a) we show the deformation of the beam for the single axis deflection system from Gooch&Housego deflector *AODF 4090-7*. For the 1D case, the highest observed deflection angle was 30° for which we offset the minimal voltage (1.47V). Additionally, in Figure S2b) we show the power distribution along the deflected angles, revealing a variation of 30%. Such variation arises from the decreasing diffraction efficiency versus diffraction angle, i.e. lower performance toward the periphery of the sample. The losses are not only limited by the MS but also by the diffraction efficiency of the AOD. After optimization of the 1st order by adjusting the incident input, we can recover about 63% of the incoming beam intensity on the [1,1] AOD deflected beam, as illustrated in figure S2c). The remaining 37% is leaking principally through the 0th order and secondly to other higher orders. These undesired optical signals are discarded from the measurements using a beam blocker placed between the AOD and the MS. The overall loss of the deflection module is summarized in figure S2d), resulting in a value of around 4dB. Additional loss of 1.5dB should be considered for 2D scanning.

S3. Calibration of 2-axis scanning

Additional calibration procedures have been realized to address 2D deflection angles, generalizing the curve in Figure S1c). To implement any arbitrary beam scanning, as illustrated in figure 3c), we fabricated a MS with radially symmetric phase profile. Any beam impinging onto the MS will thus behave according to the calibration curve in figure S1c) but by replacing linear coordinate by radial coordinate. The angular position of the beam on the MS plane, recorded by the angle θ_{MS} as showed in figure S3a) and Figure 1, defines the deflection angles (θ_t, φ_t) in Eq. 3. For accurate calibration, we assume that each point on the MS plane can be translated into a given voltage V_x and V_y, applied into the AOD driver as shown in fig. S3a). Converting the voltages V_x, V_y into polar coordinates, we define r and θ_{MS}, given by:

$$r = V_x^2 + V_y^2$$
$$\theta_{MS} = \operatorname{atan}\left(\frac{V_y}{V_x}\right) \qquad [S1]$$

and:

$$V_x = r\cos(\,\theta_{MS})$$
$$V_y = r\sin(\,\theta_{MS}) \qquad [S2]$$

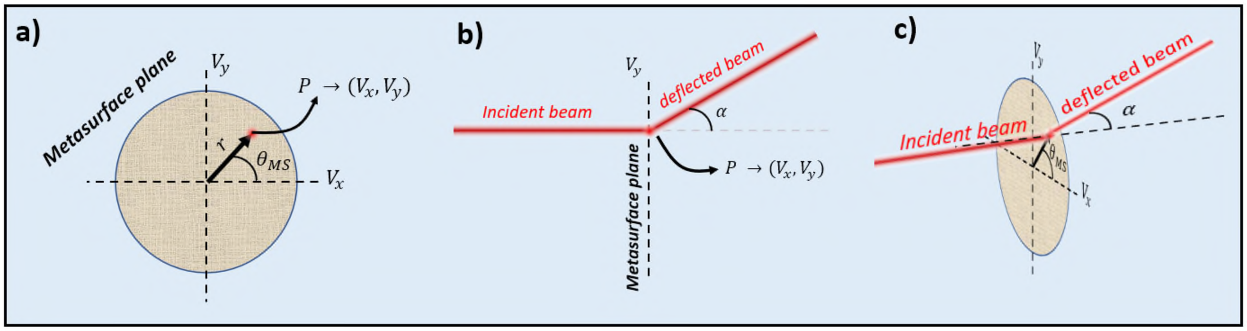

Figure S3 - Illustration of the geometries used to estimate 2D deflection calibration. a) Front view of MS plane to address the angle θ_{MS} according to the point $P(V_x, V_y)$.b) Side view, where we define a plane of symmetry perpendicular to the MS plane that will deflect the beam to an angle α. c) Perspective view illustrating the connection of the angles.

Beyond the MS, the beam is deflected to an outgoing general angle α (figure S3 b)), lying within the orthogonal plane to the MS, that acts symmetrically for any θ_{MS} as illustrated in fig. S3c). Furthermore, the angle α can be easily extracted using the calibration curve in figure S1c), depending on the point r. By associating the equivalent spherical coordinates (azimuthal θ and elevation φ), we obtain:

$$\theta_{MS} = \operatorname{atan}\left(\frac{\tan\varphi}{\sin\theta}\right) \qquad [S3]$$
$$\alpha = \operatorname{acos}(\cos\varphi\cos\theta)$$

Similarly,

$$\theta = \operatorname{atan}(\tan\alpha\cos\,\theta_{MS})$$
$$\varphi = \operatorname{asin}(\sin\alpha\sin\,\theta_{MS}) \qquad [S4]$$

With these expressions we can transform the scanning coordinates into a system based purely on the voltage control of the AOD driver. After the final conversion from $(\alpha, \theta_{MS}) \rightarrow (V_x, V_y)$ from equation S2 and the polynomial fit of the curve on figure S2c), we obtain the 2D calibration curves presented in Figure S4.

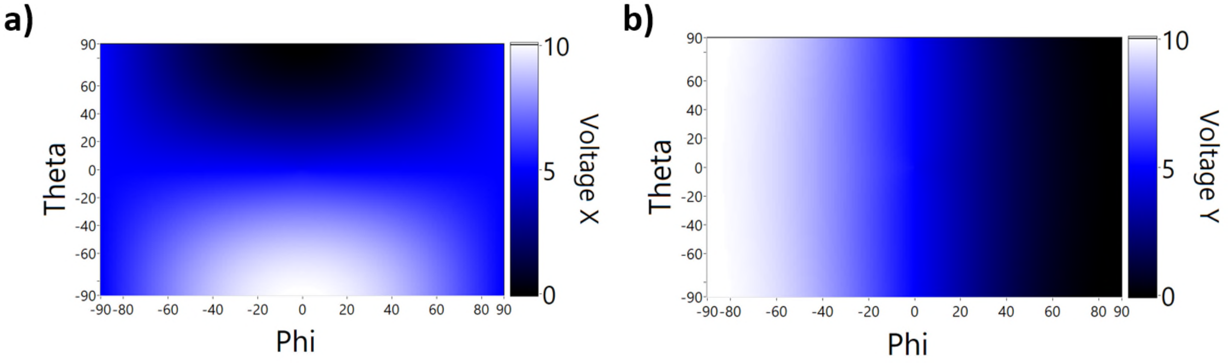

Figure S4 – Calibration maps *for both voltage X in* ***a)*** *and voltage Y in* ***b)****. These maps are used to associate a given point in the azimuthal θ and elevation ϕ angles to the voltages to be applied into the AOD driver. Such scheme allows us to perform any arbitrary scan as illustrated in figure 3****c)****.*

S4. Beam modulation speed and imaging quality

Fast scanning over the limits of AOD brings unwanted beam distortion leading to poor imaging quality, low resolution, and bad beamforming. The beam modulation speed using an AOD is intrinsically limited by the transit time τ defined by[1]:

$$\tau = \frac{D}{v_\alpha} \qquad [S4]$$

Where D is the beam diammeter (~3mm) and v_α is the acoustic velocity (650m/s). The AOD used in our experiments gives a nominal transit time of $15.4\,ns$ and a nominal scanning frequency of 216 kHz. To further investigate the limit in imaging speed, we firstly estimate the maximum beam deflecting frequency at which we can retrieve a good image quality. To test this scanning speed we performed aback and forth 2-repointing experiment. We defined the threshold frequency as the one for which the repointing signal drops to around 50%. The measurements lead to the observation of frequencies of 6 MHz (blue curve) for the 2D AOD and around 10MHz for 1D AOD beyond the estimated transit time from equation S4. In this regime of fast scanning, small beam deformations are expected and to further investigate the image quality, we performed several intensity imaging tests of a USAF target (figure S5a)) located at 1m away from the source. We observed no image degradation for scanning frequencies lower than 3.33MHz. As shown in the lower row in figure S5c), beyond this frequency, the resolution is drastically reduced indicating that the requested scanning exceeds the transit time inside the AOD and therefore beam deformation is introduced.

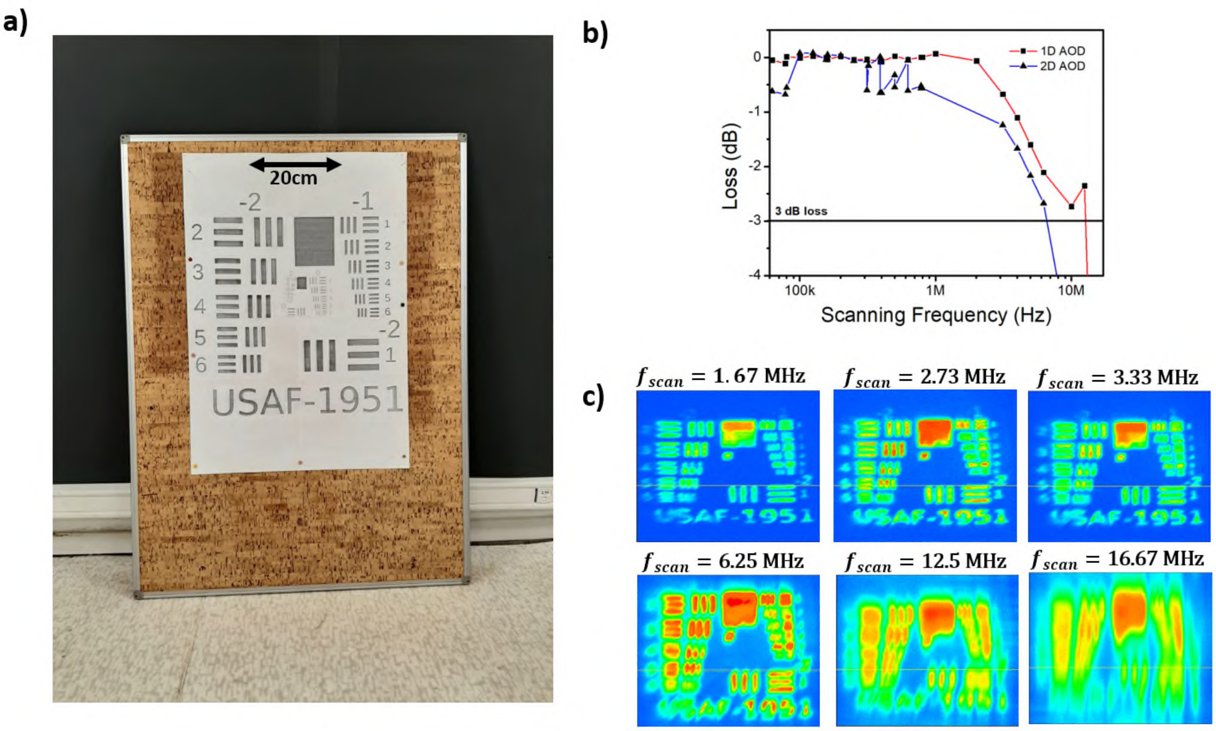

Figure S5– Beam speed and image quality. ***a)*** *Picture of the target test used to evaluate imaging quality as a function of the scanning frequency.* ***b)*** *Bandwidth response of the scanning system for 1D deflector (blue) and 2D deflector (red). The blackline on the bottom of the graph represents the 3dB threshold point where the amplitude decreases over 50%.* ***c)*** *LiDAR Intensity images showing decreasing image quality. (upper panel) image is still recovered with good resolution (lower panel) degradation of the image as the scanning moves beyond the 3dB threshold.*

S5. Beam divergence characterization

The setup illustrated in figure 1a) and 1b), contains a scanning lens which ensures that the beam waist is the same at the focal plane regardless the wave vector. However, the beam impinging on the MS has a physical size, and thus overlays only a certain number of meta-atoms. The section of illuminated meta-atoms is function of the beam size at the focal region. The MS introduces beam divergence that thus depends on the beam size. To analyze the effects of the beam spot on the MS on transmitted beam divergence, we measured the beam diameter (using a CCD camera) as a function of the ranging distance, as illustrated in figure S6a). We collected the beam profile intensity on imaging planes located at various increasing distances from the MS, as show in figure S6b). We extracted the beam profile by defining the central point of the beam and by performing an integration in the angular axis using polar coordinates. The extracted curves and their respective Gaussian fits are illustrated in figure S6c). These data were used to estimate the divergence by performing a linear regression of the beam waist as a function of the distance as illustrated in Figure S6d).

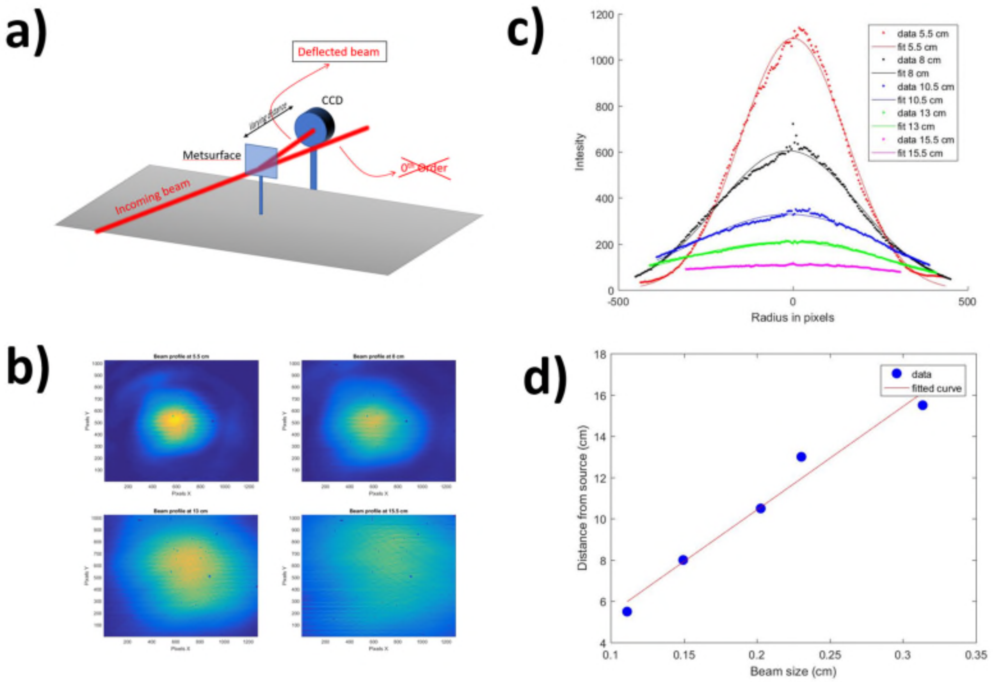

*Figure S6 – **Beam divergence setup and measurements for the MS with 1mm of diameter**. In **a)** we illustrate the experimental setup employed. We analyze the beam profile for every distance Z from the MS by using a CCD and the results for Z= [5.5,8,13,15.5]cm is illustrated in **b)**. **c)** For each distance we extract the radial profile from the center of the beam and fitted using a Gaussian function. In **d)** we show the linear regression performed given a beam divergence of 4.5°.*

Such experiments and analysis were performed for three different MS diameter sizes (1mm ,2mm and 3mm), while keeping the same MS phase function. Intensity data of the beam profile for different samples are presented at figure S7a) and the compilation is displayed in figure S7b). In fact, and as expected, by increasing the MS size, and thus decreasing the spatial variation of the phase profile for a fixed beam spot, one reduces the divergence of the deflected beam. Considering ADAS and robotic vision applications, a divergence value of 0.25 degrees allows resolving small objects located far away (~200m), (see the patterned dot in figure S7 b)). The graph indicates that such performances are obtained by further increasing the sample diameter up to 3.9mm. Note that such analysis works only when considering the setup including a scanning lens with an input beam of 3mm into the AOD. Any modification of the beam along the setup, including a different choice of optics, would result to different divergence. Furthermore, by changing the optical system, we must reconsider all the calibration curves presented in the figure S4.

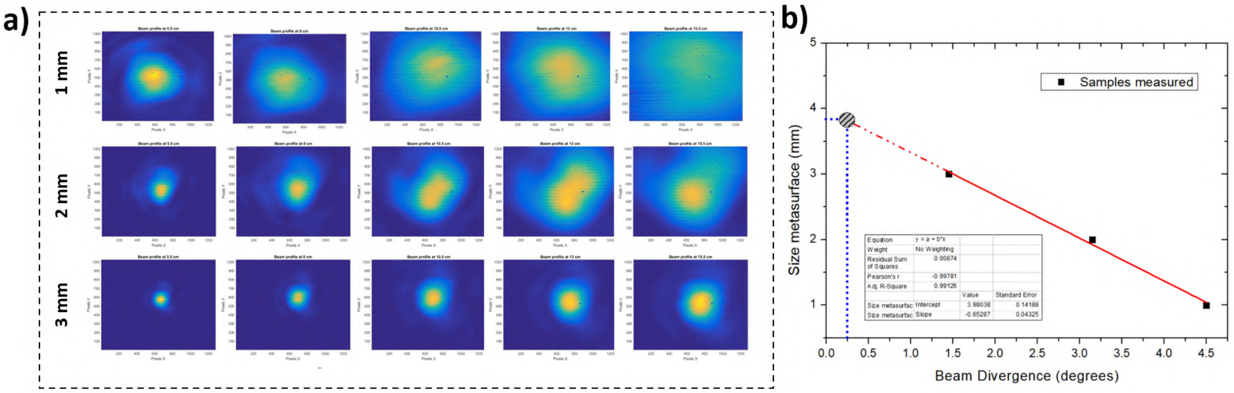

***Figure S7 – Beam profile and divergence**. a) beam profile to estimate the beam divergence over different ranging z-distances. We performed a Gaussian fit of the radial profile and extracted the FWHM as a function of the distance. Performing a linear regression, we estimated the beam divergence. Such measurements were investigated under different MS diameters and the respective divergence extracted for each sampla, as illustrated in b). Note that a performant LiDAR requires a divergence as low as 0.25 degrees, which would be accessible with a MS of 3.9mm diameter.*

S6. Data generation, acquisition, and analysis

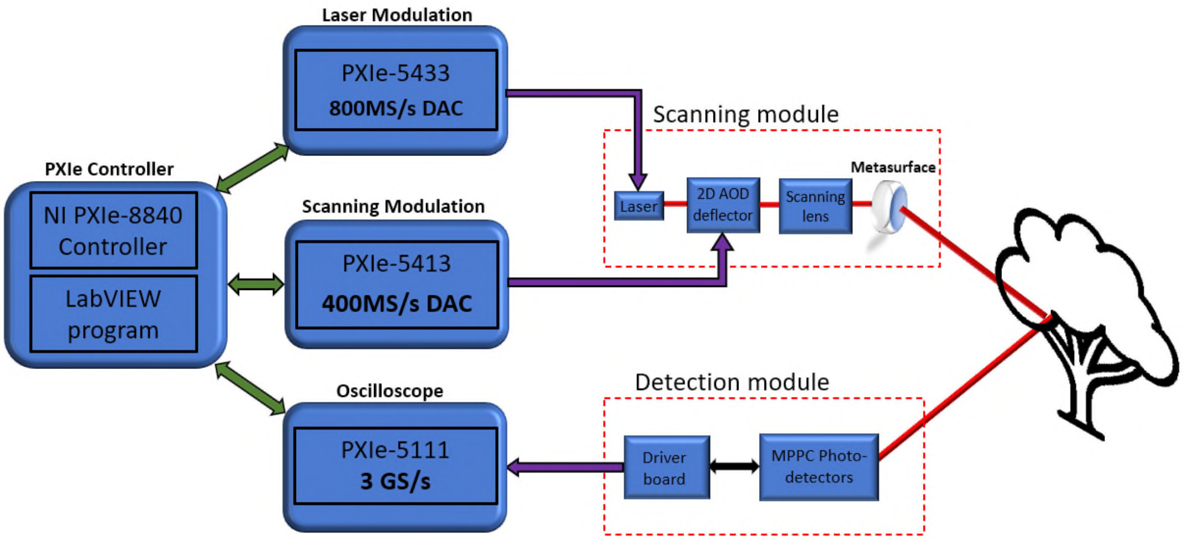

*Figure S8 – **Schematics of the logical path to retrieve ranging image.** A synchronous PXi system assisted by a graphical user interface in LabView controls different modules to perform various tasks. The scanning modules consists of a laser modulation system and a 2-channels scanning modulation to create single ToF pulses and scanning directions, respectively. The detection module is coupled to an oscilloscope module that acquires, stores and fetches data used for imaging reconstruction.*

To electronically drive the whole system presented in this work, we employed a synchronized PXIe system consisting of different controllers built-in a chassis. The chassis is the foundation of the system, mounted on a rack-type container, providing a common clock and featuring high-speed data-transfer and high speed inter-communication between the modules. This scheme gives PXIe's modules the ability to reach high level of synchronization and low jitter needed for LiDAR systems. The employed modules for the development of the LiDAR presented herein are: (i) a controller - containing the logical part (processor, RAM memory, hard drive), (ii) two frequency generator (FGEN) cards - one to control the scanning, and

the other to control the laser, and (iii) an oscilloscope (SCOPE) featuring an analog-to-digital-converter (ADC) to fetch the voltage data from the photodetector. A detailed architecture scheme summarizing the features and the models of all the elements of the LiDAR is provided in figure S8. The module used to drive the laser source is a PXIe-5433 FGEN, generating arbitrary waveforms of +/-12V amplitude, with a maximum sampling rate (SR) of 800MS/s. For controlling the scanning, we employed a PXIe-5413, using a SR of maximum 200MS/s. Finally, for the SCOPE module we used the PXIe-5111, featuring dual channel operation with a shared SR of up to 3GS/S. The use of a double-channel is crucial for enabling the multi-zone imaging capabilities presented in figure 4c). The SCOPE SR defines the depth resolution of the LiDAR: *i.e.*, at 3GS/s, the smallest measurable time is 0.33ns, corresponding to a minimal depth precision of $5\ cm$. To ensure that synchronization remains locked over time and avoid any temporal drift, a SR has been used for each module. Each module's SR has been selected to be an integer multiple of the other modules SR to always ensure an integer number of time synchronous samples. The main constraint is to keep the SR of the SCOPE module as high as possible to assure the maximum ranging precision as discussed in the main paper. In summary, to highly leverage the most performant SRs, we must use 300MS/s for the laser driving FGEN and 150MS/s for the scanner driving FGEN since 3GS/s in the SCOPE is multiple of both these SR.

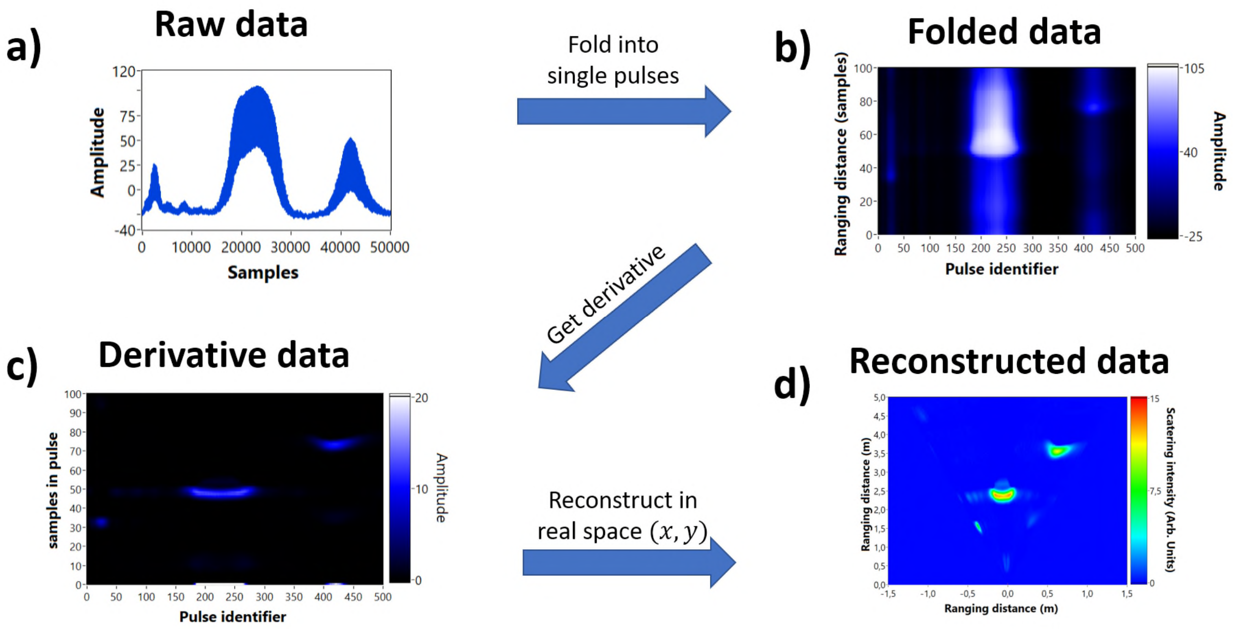

Figure S9 – ***Step-by-step of data analysis and imaging retrieving algorithm used in this work.*** *In a) The whole raw data is collected in a single shot measurement corresponding to single ToF pulses from every single line direction of the scan. In b) the data is folded to tag every single direction corresponding to ToF pulses. In c) we perform the derivative of the folded data to find the rising time corresponding to the ToF. Finally in d), we reconstruct the data considering the calibration curve.*

For 2D imaging (1D scanning), data treatment was performed in several steps. After raw signal collection, displayed in Fig S9 a), we folded the data into a matrix (figure S9b)) containing $N \times M$ points, where N is the total number of samples within a single ToF pulse and M indicates the number of resolvable spots in the LiDAR imaging, accounting for the number of pixels. Subsequently, we calculated the derivative of every single M ToF pulses for edge information, as shown in figure S9c). The data is then reconstructed (figure S9d)) by extracting both the intensity and the ToF of every direction, considering the calibrated

angles discussed in the last section. Such approach can be extended for 3D imaging by adding another dimension on the scanning.

S7 – MHz frame rate, 1D scanning

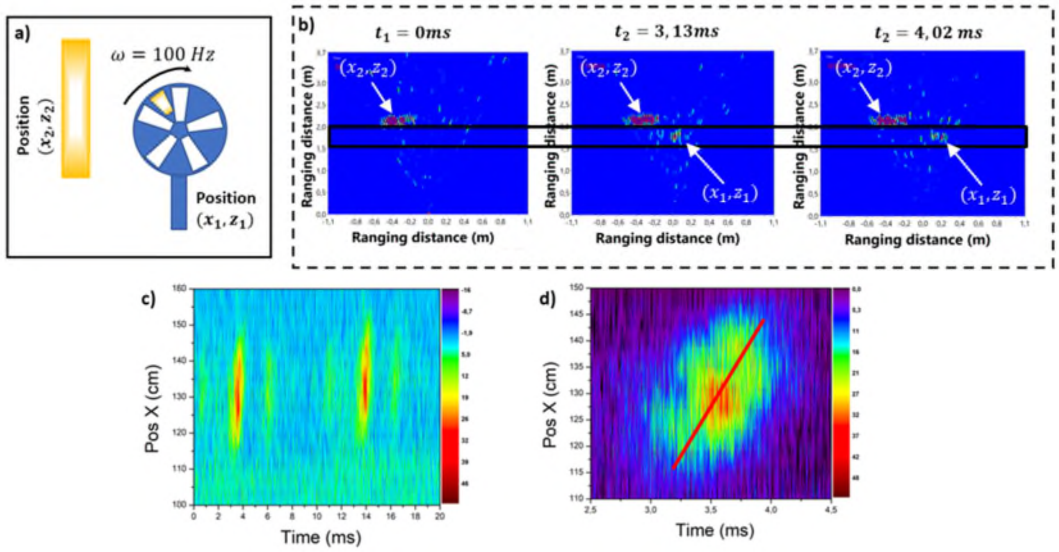

*Figure S10 - **Measurement of MHz scanning.** In a) we show the concept of the experiment: an optical chopper with an angular velocity of $100\ Hz$ was place 50cm in front of a high reflective ribbon in the back. A high reflective tape was also placed in one side of the chopper, represented as a golden tape. In b) left we illustrate the ranging image frame cut at the beginning of the experiment at $t = 0\ ms$ which shows the back reflected ribbon at the ranging position of 2m; in the middle, at $t = 3.13\ ms$ we show the moment where the tape passes through the scanning position of the beam at 1.5m represented by a small spot. The plot on the left corresponds to the moment when tape passes close to the end of the scanning line at $t = 4.02ms$. In c) We show a separation of the position 1.5m in the ranging axis which isolates the moving signal only. In graph there are two peaks at $t = 3.5ms$ and $t = 13.5ms$ showing a period of 10ms which corresponds to the 100Hz angular speed. In d) we show a detailed highlight on the first oscillation, the red curve is an illustration of the ribbon speed of around 1400Km/h.*

To demonstrate the ultimate imaging performance of our system, we performed LiDAR imaging of a moving object in two-dimensions (X-Z) by using a single line scanning AOD (GH AODF-4090-7). The fastest available object found in our lab to perform this experiment is a turning optical chopper that was placed on the optical table in the vicinity of a static high reflective ribbon to recover ranging measurement with dynamical and static bodies, as indicated in Figure S10a. The golden tape, at the position (x_1, z_1), represents a moving reflective tape placed on one section of the optical chopper (dynamic object), and the reflective ribbon in the back represents the static object, situated at the position (x_2, z_2).The spinning frequency of the optical chopper was set to the maximum angular speed of $100\ Hz$ allowing fast response characterization. In figure S10b) we present three frame shots taken at different times to illustrate LiDAR's ability to capture moving objects. Particularly, here this dynamic imaging ability is related to the different positions of the reflective tape across the angular scanning range of the LiDAR scene. The image on the left, at $t = 0ms$, corresponds to the instant where the tape does not cross the LIDAR scanning line, in which we observe only the back reflective ribbon located 2m away from source. In the middle panel, at $t = 3.13ms$, the reflective tape passes thought the line of sight of the laser, as indicated by a red amplitude peak around 1.5m away from the source. Finally, at $t = 4ms$, the reflective tape on the chopper moves to the right side of the image, completing the rotation cycle across the LIDAR sight range.Figure S10c) shows the space-time $(x - t)$ LIDAR images obtained after integrated z-pixels around

1.6m, highlighting the movement of the reflective tape. We clearly observe two diagonal peaks delayed by10 ms andcorresponding to the frequency of 100Hz of the oscillation. Furthermore, we can monitor the presence of two small peaks at the side, which correspond to some spurious reflection of the edges of chopper's blades. Isolating the (x, t) zoomed image section at 3.5m, in Figure S10d), we reveal the displacement of the tape along the x direction during a single period of rotation only. For the sake of illustration, we calculated the linear speed of moving tape by placing a diagonal red line on the image, leading to a displacement speed of 30cm/1ms, or ~1400 Km/h. These results demonstrate the ability of the high frame rate imaging LiDAR system to observe fast, even supersonic, moving objects.

S8. Details on the three dynamic beam scanning experiments

The results presented in the section of high speed and time-series imaging summarized in Figure 5, were repeated for three different configurations according to specifications presented in Table 1. For each measurement we kept the same ranging limit, and we estimated the size of the reflective tape on the chopper. In the first measurement, presented in figure S11a), we setup the imaging to scan at 16.66MHz, aiming to larger number of pixels, 150X150, resulting in a framerate of 741fps. Even with many pixels (scanning points), the estimated size of the tape was slightly bigger than the expected one. This evidence corroborates with the intensity maps showed in figure S5c). Due to the high scanning speed, beyond the 3dB limit, the image quality degenerates. We could still retrieve the dynamics of the rotating chopper but with lower temporal resolution. On the other hand, the 2nd experiment (figure S11 b)), where we kept the scanning speed at 5MHz and we reduced the number of pixels by 70X70, enables retrieving the correct tape size, while keeping a good temporal resolution. Finally, in figure S11c), where the scanning was set to 16.66MHz and the number of pixels was 70X70, we achieved the best temporal resolution of 3kpfs, see central column. All the three measurements are dynamically showed in Supplementary GIF1-3. The optimal situation must set after decide the compromise between resolution and scanning speed.

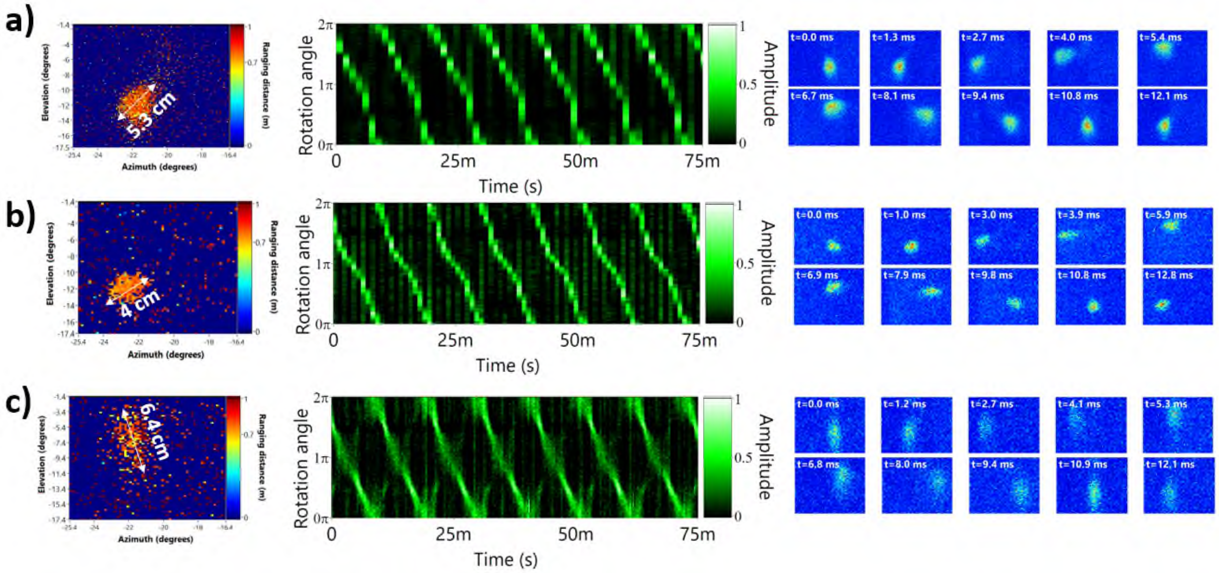

Figure S11 - Full Dynamic measurements. Details of experimental conditions for each row are summarized in table 1 for a) 741 fps, b) 1.02kfps and c) 3.4 kfps.

Experiment	Framerate	Number of pixels	Time step	speed	Maximum distance	Speed	Total Frames
**1**	741 fps	150X150 pixels	1350µs	16.67 MHz	8.99m	92.03Hz	63
**2**	1020 fps	70X70 pixels	980 µs	5MHz	29.97m	92.9Hz	87
**3**	3401 fps	70X70 pixels	294 µs	16.67 MHz	8.99m	93.2Hz	290

Table 1 – Parameters of the three experiments to measure the rotation speed. First column is the experiment number. Second column is the number of frames-per-second used in the experiment. Third column summarizes the image resolution. Fourth column represents the time-resolution of each frame collected. Fifth column represent the scanning speed and laser repetition rate used, whose correspondent ranging image is shown in the sixth column. Seventh column is the measured chopper speed, from the curve in fig 7a(bottom). Eight column corresponds to the number of acquired frames using these parameters, such value is limited by the scope card used.

S9. Metasurface fabrication

The fabrication of the various MS used in these experiments has been realized following two independent processes. For the small components of diameter 1,2 and 3mm we followed our standard GaN metasurface process, consisting of growing a GaN layer on a double side polished (111) Sapphire substrate using Metal-organic chemical vapor deposition (MOCVD) reactor. MOCVD provides accurate thickness control, as well as large-scale uniformity of the GaN layer. We then followed up the fabrication process by employing electron beam lithography, considering a Hydrogen silsesquioxane (HSQ) resist spin-coated onto the GaN. After exposition, the resist pattern is used as an etching mask for the RIE process. Following the GaN RIE etching, the leftover of resist was removed using chemical native oxide removal by dipping the patterned films in a BOE etch. The results of the fabrication are shown in figure S12.

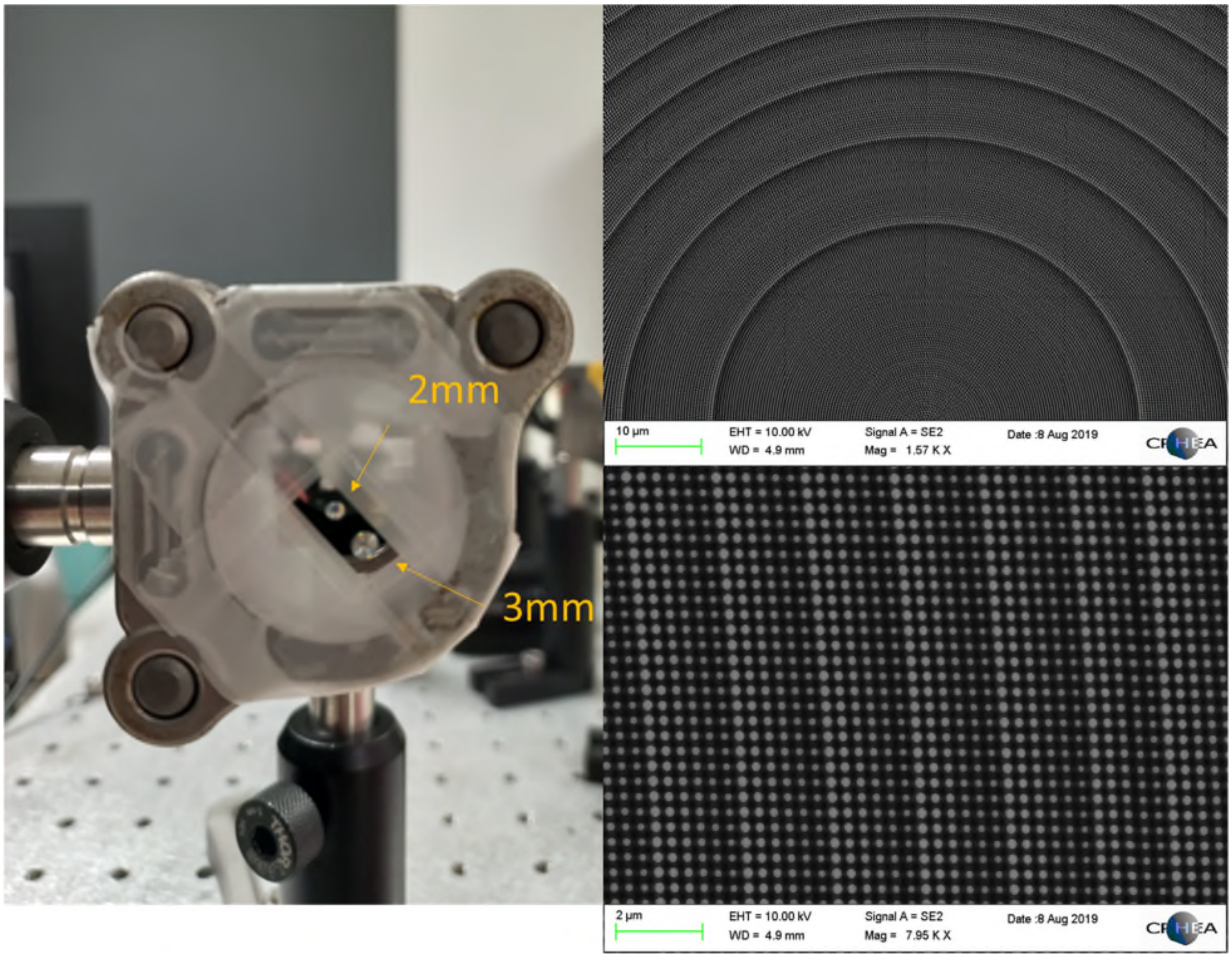

Figure S12 - Sample details. In a) is a photography of the sample containing two MS deflectors. b) SEM image of the nanopillars showing the size variation to achieve deflection and c) detail on a few nanopillar unit cells to illustrate the phase gradient periodicity;

The large-area metasurfaces used in dual FoV imaging experiments (see Figure 5) have been fabricated using nano-imprint lithography (NIL). The NIL process starts with the e-beam fabrication of a large area master mold. A soft textured stamp is derived from the master mold, to implement the NIL process. Due to the different fabrication steps, the pattern of the final structure is the negative counterpart of the structure of the master mold. Here, the master mold is designed with holes in order to obtain pillars on GaN etched substrate. The fabrication process is described on figure S13. From the initial master mold, a textured stamp is fabricated, made of the inverse structure (holes in the master mold become pillars in the stamp) (step 1) A sacrificial PMMA layer is coated on a GaN/Al_2O_3 substrate. A proprietary SiO_2 sol-gel resist is then coated on the PMMA layer and patterned with holes, using the previously fabricated textured stamp (step 2-3). The objective is then to open both resists, to reach the GaN layer of the template. A first etching process is performed using a chlorinated plasma during 5min50s to etch the residual SiO_2 sol-gel layer (layer remaining at the bottom of the holes), to reach the PMMA layer. Then, the PMMA is etched during 1min20s. These two etching steps have been implemented by ECR-RIE (step 3-4). Once the pattern is opened, meaning the holes are directly on the GaN layer, a 50 nm think nickel

layer is deposited (step 5), on top of the patterns and at the bottom of the holes. The remaining hole structure is then lifted-off: the substrate is immerged in a solvent of the PMMA resist and all resists are removed from the substrate leaving only nanometric nickel disks on the substrate (step 6). Following the lift-off, the GaN template is etched with chlorinated plasma to obtain the desired pillars structure, using the nickel disks as an etching mask (step 7). Finally, the nickel mask is removed by a wet acid etching (step 8).

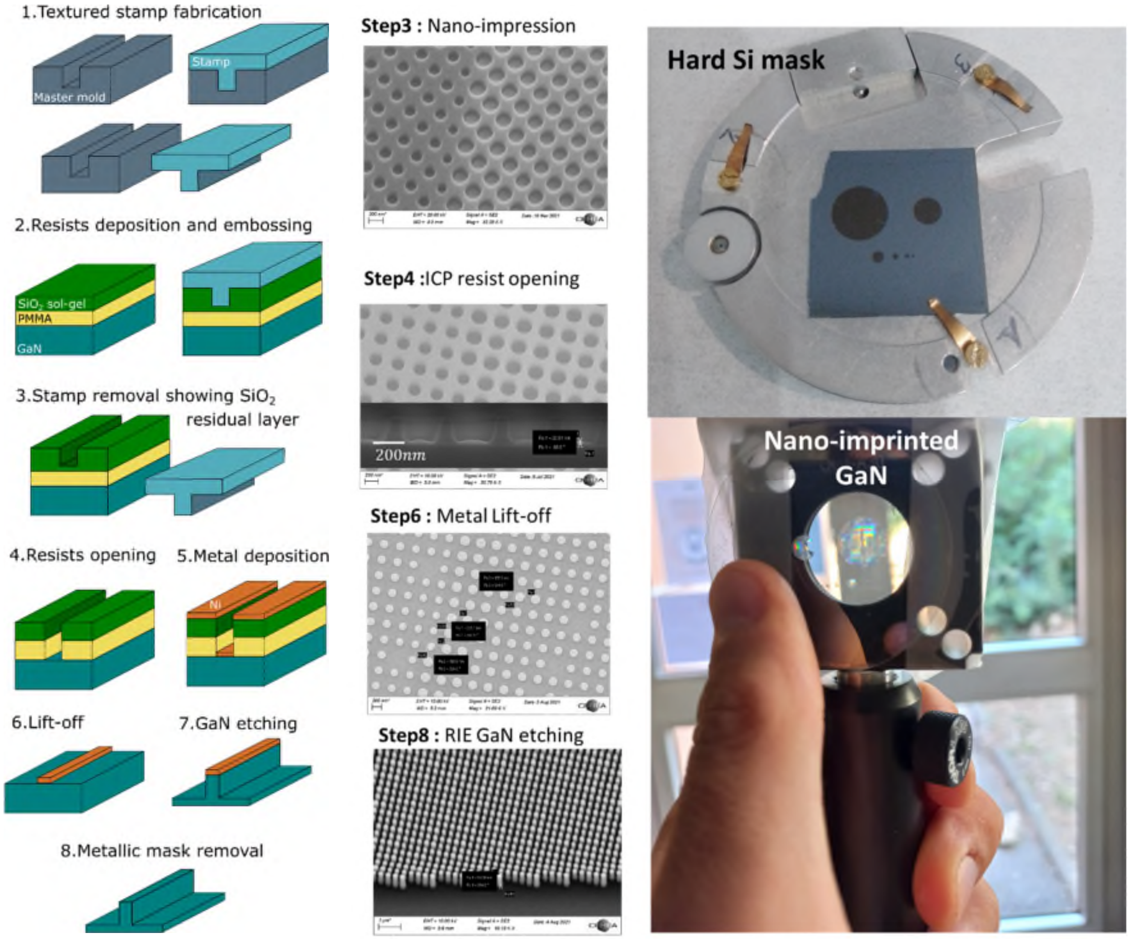

Figure S13 – Nanoimprinting process to create large area MS used for double zone imaging experiments.

S10. Number of resolvable spots

The number of resolvable spots N depends on the divergence of the output beam and the maximum FoV achievable. In the demonstrated system, as a proof-of-concept, we measured 1.8 degrees of divergence (figure S1b)) giving $N{\sim}83$. In a typical acousto-optic deflector, the number of resolvable spots is around 500, giving thus the upper bound of the resolution. The principle of beam deflection relies on the generalized Snell's law, given by:

$$\sin(\theta_t)n_t - \sin(\theta_i)n_i = \frac{\lambda}{2\pi}\frac{d\Phi}{dr}$$

In this expression, θ_t and θ_i represent the transmitted and incident beam arriving on the metasurface and $\Phi(r)$ represents the phase function to be encoded in the sample. The current work used a quadratic phase function, which corresponds to a gradual increasing of the amount of linear momentum (quadratic phase) given by $\Phi(r) = \frac{\pi}{\lambda}\frac{r^2}{r_{max}}$, where r_{max} is the size of the metasurface. Such a phase function results in a divergence of about 1–3 degrees using a 2 mm metasurface and a 50 µm beam size. Moreover, each laser beam, defined by a factor M^2, is itself an aperture, *i.e.*, it has an intrinsic divergence due to diffraction optics that is given by:

$$\Delta\theta_d = \frac{M^2\lambda}{\pi\, w_0}$$

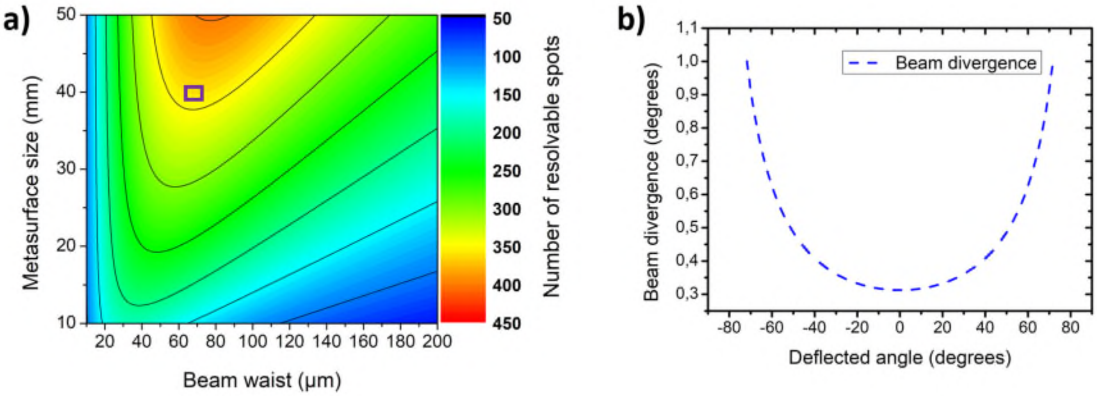

Figure S14: ***Number of resolvable spots****.* ***a)*** *Parametric map of number of resolvable spots in function of beam waist and metasurface size, the inset square represents the point* $w = 70\mu m$ *and* $= 4cm$ *.* ***b)*** *divergence of the beam in function of deflected angle from inset in a).*

This expression also indicates that, the greater the size of the beam w_0, the lower the divergence of the collimated beam and higher is the number of resolvable spots.

Relying on the above equations, we estimate the combination of the smallest beam size and the metasurface size to maximize the number of resolvable spots by considering the intrinsic beam divergence, for the actual applied phase function only. A small parametric search (in figure S8a)) has shown an increase (4-5X factor) in N from 86 to 400 using a 4cm metasurface and 70µm beam size, which seems achievable using a similar scanning lens. Furthermore, it is important to address that the divergence can increase significantly for high deflection angles. For instance, Figure S8b) illustrates the change in the beam divergence as a function of the deflected angles (position of the beam in the metasurface), indicating a variation from 0.3 degrees up 1 degree for 75 degrees deflection. This effect can be taken into consideration and reduced by employing other advanced phase functions

References

1. Römer GRBE, Bechtold P. Electro-optic and Acousto-optic Laser Beam Scanners. *Phys Procedia*. 2014;56:29-39. doi:10.1016/j.phpro.2014.08.092
