# Supplementary material for: Metasurface-enhanced light detection and ranging technology
Source: Nat Commun. 2022 Sep 29;13:5724. doi: 10.1038/s41467-022-33450-2 (PMC9523074; doi:10.1038/s41467-022-33450-2)
Supplement: Supplementary file 3 — Description to Additional Supplementary Information [file 41467_2022_33450_MOESM3_ESM.pdf]

Description of Additional Supplementary Files

Supplementary Video V1 - In-lab demonstration of 150X150 Field of view.

Supplementary Video V2 - Beam projection to demonstrate random-beam access mode.

Supplementary Video V3 - Demonstration of real time imaging at low frame rates.

Supplementary GIF1 - rotating chopper imaged with a framerate of 740fps

Supplementary GIF2 - rotating chopper imaged with a framerate of 740fps

Supplementary GIF3 - rotating chopper imaged with a framerate of 740fps
