# Supplementary figures and images for: Metasurface-enhanced light detection and ranging technology
Source: Nat Commun. 2022 Sep 29;13:5724. doi: 10.1038/s41467-022-33450-2 (PMC9523074; doi:10.1038/s41467-022-33450-2)

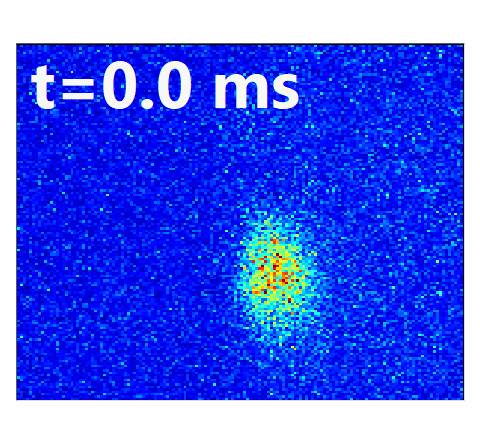

Supplement: Supplementary file 4 — Supplementary GIF 1 [file 41467_2022_33450_MOESM4_ESM.gif]

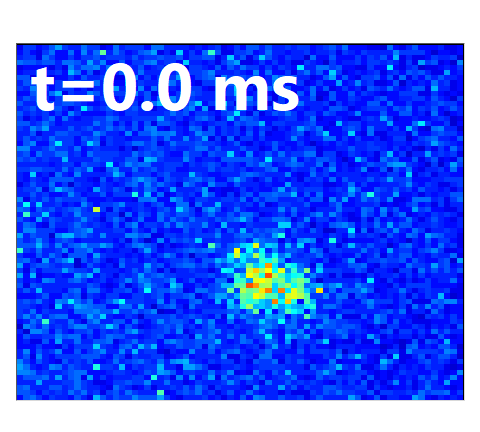

Supplement: Supplementary file 5 — Supplementary GIF 2 [file 41467_2022_33450_MOESM5_ESM.gif]

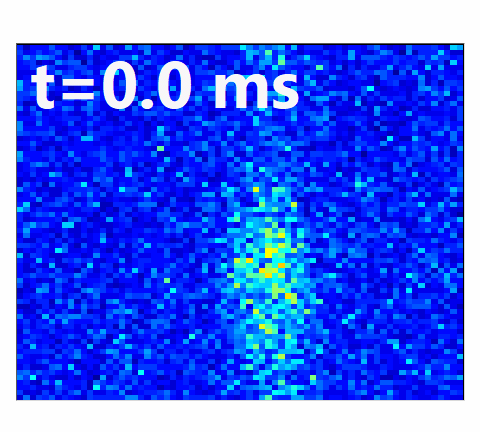

Supplement: Supplementary file 6 — Supplementary GIF 3 [file 41467_2022_33450_MOESM6_ESM.gif]
